# Prevalence and determinants of gestational diabetes mellitus in Africa based on the updated international diagnostic criteria: a systematic review and meta-analysis

**DOI:** 10.1186/s13690-019-0362-0

**Published:** 2019-08-06

**Authors:** Achenef Asmamaw Muche, Oladapo O. Olayemi, Yigzaw Kebede Gete

**Affiliations:** 1 Department of Obstetrics and Gynaecology, College of Medicine, Pan African University Life and Earth Sciences Institutes, University of Ibadan, Ibadan, Nigeria; 2 Department of Obstetrics and Gynaecology, College of Medicine, University College Hospital, University of Ibadan, Ibadan, Nigeria; 3 Department of Epidemiology and Biostatistics, Institute of Public Health, University of Gondar, Gondar, Ethiopia

**Keywords:** Prevalence, Gestational diabetes mellitus, Determinants, Updated diagnostic criteria Africa, Systematic review, Meta-analysis

## Abstract

**Background:**

Gestational diabetes mellitus (GDM) is a major public health problem and threat to maternal and child health in Africa. No prior review has been conducted in Africa using the updated GDM diagnostic criteria. Therefore, this review aimed to estimate the pooled prevalence and determinants of GDM in Africa by using current international diagnostic criteria.

**Methods:**

A systematic review and meta-analysis was conducted by comprehensive search of the published studies in Africa. Electronic databases (PubMed, Scopus, Cochrane Library, EMBASE, Google Scholar, CINAHL, Web of Science, Science direct and African Journals Online) were searched using relevant search terms. Data were extracted on an excel sheet and Stata/ SE 14.0 software was used to perform the meta-analysis. Heterogeneity of included studies were assessed using *I*^2^ and Q test statistics. I^2^ > 50% and Q test with its respective *p*-value < 0.05 were suggestive for the presence of a significant heterogeneity. Publication bias was assessed using the Egger‘s regression test and funnel plot. Subgroup and sensitivity analyses were done. A random effects model was used to estimate the pooled prevalence of GDM and odds ratio (OR) with 95% confidence interval (CI).

**Result:**

A total of 23 studies were included in the final analysis. The pooled prevalence of GDM in Africa was 13.61% (95% CI: 10.99, 16.23; I^2^ = 96.1%), and 14.28% (95% CI, 11.39, 17.16; I^2^ = 96.4%) in the sub-Saharan African region. The prevalence was highest in Central Africa 20.4% (95% CI, 1.55, 38.54), and lowest in Northern Africa 7.57% (95% CI, 5.89, 9.25) sub- regions. Overweight and obesity, macrosomia, family history of diabetes, history of stillbirth, history of abortion, chronic hypertension and history of previous GDM had positively associated with GDM.

**Conclusions:**

The prevalence of GDM is high in Africa. Being overweighed and/or obese, ever had macrocosmic baby, family history of diabetes, history of stillbirth, history of abortion or miscarriage, chronic hypertension and history of previous GDM were factors associated with GDM. Preventing overweighed and obese, giving due attention to women having high-risk cases for GDM in pregnancy are strongly recommended to mitigate the burden.

**Systematic review registration:**

PROSPERO (2018:CRD42018116843).

**Electronic supplementary material:**

The online version of this article (10.1186/s13690-019-0362-0) contains supplementary material, which is available to authorized users.

## Background

The World Health Organization (WHO) defined Gestational Diabetes Mellitus (GDM) as “any degree of glucose intolerance with onset, or first recognized during pregnancy” [1]. GDM occurred by the increased severity of insulin resistance as well as an impairment of the compensatory increase in insulin secretion during pregnancy [2]. It causes a diverse range of adverse maternal and neonatal outcomes [3] and it is a threat to maternal and child health [4].

The global prevalence of GDM varies widely from 1 to 28% depending on population characteristics, screening methods, and diagnostic criteria [5]. The International Diabetes Federation (IDF)-2015 report showed that about 16.2% of women had some form of hyperglycemia during pregnancy, of which GDM shares about 85.1% of the load [6]. A review revealed the prevalence varies from 5.4% in Europe [7] to 11.5% in Asia [8]. Similarly, the IDF report indicated that there were regional differences in the magnitude of hyperglycemia during pregnancy, for instance, the South-East Asia region had higher (24.2%) as compared to 10.5% of the Africa Region. In addition, the majority (87.6%) of GDM accounts in low and middle-income countries, where access to maternal care was often limited [6].

A review indicated that the occurrence of GDM in sub-Saharan Africa was 14% [9] and the Middle East and North Africa ranged from 8.4 to 24.5% [10] though the study used different screening and diagnostic criteria which masked the true prevalence. Studies also showed that GDM also sees varied among African regions to a certain extent, for instance, East Africa (6%) [11] and West Africa (14%) [12]. Moreover, there were variations within the same sub-regions, like in Rwanda (8.3%) [13], Tanzania (5.9%) [11], and Ethiopia (3.7%) [14]. This disparity in GDM prevalence rate may be due to differences in diagnostic criteria [15–17], screening strategies [9, 18, 19], and population characteristics [20].

Recently, the highest rise in the incidence of obesity, diabetes and other non-communicable diseases are expected to occur in low and medium income countries (LMICs) especially in Africa. It was one of the challenging health problems of sub-Saharan African countries [21, 22].

The approach to screening and diagnosis of GDM around the world has historically been shrouded in controversies and the use of different diagnostic criteria results the different prevalence of GDM.

Lack of uniformity in the protocols for diagnosis vary not only in-between countries, but also within countries and makes it difficult to compare the prevalence of GDM between and within countries. However, in 2013, WHO revised its recommendations for the diagnosis of GDM taking into cognizance the issues raised by the International Association of Diabetes in Pregnancy Study Groups (IADPSG) recommendations [1, 23, 24]. The WHO 2013 modifications along with common diagnostic criteria for GDM by 2013 American Diabetes Association (ADA) [25]. Though, in the last five years, the diagnostic criteria have been changed and there is no known overall prevalence and associated factors of GDM after the change in Africa.

The previous review included studies only in sub-Saharan African countries with lack of uniformity in screening methods, definition, and diagnostic criteria for GDM makes it difficult to compare the prevalence of GDM between and within countries and point out the true pooled prevalence. Furthermore, the review did not include studies conducting by using updated or current diagnostic criteria (WHO 2013), and did not report findings on risk factors for GDM based in the new diagnostic criteria (WHO 2013) [9] and again review without meta-analysis included few studies did not investigate the sources of heterogeneity between the studies and did not report findings on risk factors for GDM [26]. Inconsistent findings were noted among studies conducted in Africa regarding the magnitude as well as factors associated with GDM [9, 15, 26].

There has been no review on the overall prevalence of GDM in Africa and in the sub-regions based on the updated diagnostic criteria. Therefore, the aim of this meta-analysis is to estimate the prevalence of GDM in a broader scope including the countries across Africa using the pieces of evidence from those studies conducted by using the updated diagnostic criteria for GDM. In addition, we also examine the risk factors for GDM among the African populations.

The findings of this study would underscore the importance and urgency of scaling-up GDM screening and its management throughout Africa. Moreover, understanding the prevalence and determinates of GDM in Africa may provide evidence on how interventions should be targeted to reduce the magnitude of the problem, to improve maternal and child health, and halt the burden of GDM in the sub-regions of Africa.

Therefore, We conducted a systematic review and meta-analysis to determine the prevalence and determinates of GDM in Africa, using pieces of evidence based on the updated and the current international GDM diagnostic criteria.

## Materials and methods

### Protocol and registration

The present review was registered with PROSPERO(2018:CRD42018116843) [27] and conducted according to the Preferred Reporting Items for Systematic Reviews and Meta Analyses (PRISMA) [28]. A protocol was developed during the planning process.

### Study design and search strategy

A systematic review and meta-analysis was conducted using published articles on the prevalence and associated factors of GDM in Africa with the updated international diagnostic criteria. The databases used to search for studies were PubMed, Scopus, Cochrane Library, EMBASE, Google Scholar, CINAHL, Web of Science, Science direct and African Journals Online (AJOL). All potentially eligible studies were accessed through this searching strategy for “*gestational diabetes mellitus* OR *hyperglycemia in pregnancy* OR impaired *glucose tolerance* OR gestational *hyperglycemia*” AND “name of Africa countries” were used separately and in combination of the Boolean operators terms “OR” and “AND” as necessary. Likewise, terms like “determinant factors OR determinant variables OR associated factors” were used in combination with the above search terms. The search was also made by combining the above search terms with the names of all countries included in Africa and sub-region of Africa. A combination of expanded search term and free-text searches were used as shown in Table 4 in Appendix 1. Then the reference lists of the retrieved studies were also followed to access for additional articles and screened for its suitability to be recruited into this review.

### Eligibility criteria

#### Inclusion criteria

Any studies in Africa that reported prevalence and risk factors for GDM and fulfilled the following criteria were entered into the analysis, including the following factors: (1) being conducted in African countries classified by the United Nations Statistics Division [29]; (2) Epidemiological studies had reported prevalence and risk factors of GDM as primary results; (3) provided the prevalence and OR with 95% confidence interval (CI) or total of participants and number of GDM events (3) Being published in English language journals from January 1, 2013 to November 26, 2018; (4) studies conducted on pregnant women regardless of gestational age, sample size and study setting; and (5) studies used the updated international diagnostic criteria for GDM diagnosis was made by using the new 2013 WHO [1] or ADA [25] or modified IADPSG [30] diagnostic criteria.

#### Exclusion criteria

If an article failed to mention any of the above inclusion criteria it was excluded. In addition, studies were excluded if they were: (1) studies with poor definition of the outcome of interest; (2) qualitative studies, review articles, case reports, and case series regardless of the number of cases, narrative reviews, conference abstracts with no full information or if authors have not responded to our inquiry on the full text, editorials, commentaries, letters to the editor, author replies, and other publications that do not include quantitative data on the prevalence and/or associated factors of GDM; (3) studies presenting contradictory/unclear quantitative measures that could not be verified with authors; (4) duplicated studies on GDM ascertainment in the same population. In the case of duplicated publications, only the study containing the most important information in the context of prevalence and ascertainment methodologies or most recent results was included; and (5) studies including GDM patients with other metabolic disorders or other non-communicable diseases (NCDs) in the same category.

#### Outcomes measurement

Gestational diabetes mellitus was diagnosis, if one or more of the following abnormality are met, fasting plasma glucose 5.1–6.9 mmol/l (92–126 mg/dl), one-hour plasma glucose ≥10.0 mmol/l (180 mg/dl), 2-h glucose 8.5–11 mmol/l (153–199 mg/dl) after overnight fasting with 75 g glucose load [1, 25, 30]. WHO endorsed the modified IADPSG criteria by 2013 [31].

#### Study selection

Relevant papers identified from the aforementioned databases and websites were imported into an EndNote X7, and duplicates were removed. Retrieved articles were assessed by two review authors with extensive experience in systematic reviews. Screening of titles abstracts and full text quality was conducted independently by two review authors (A & Y) on the bases of these inclusion criteria. Disagreement between the two reviewers was resolved by consensus or the third reviewer (O) made the decision regarding inclusion of the article in the final review.

#### Data extraction and quality assessment

The selected papers were fully reviewed and the required information for the systematic review was extracted and summarized using an extraction table in Microsoft Office Excel software. The Preferred Reporting Items for Systematic Reviews and Meta-Analyses (PRISMA) guideline were followed throughout the review and analysis processes [32]. The study quality or risk of bias was assessed using the adopted a risk of bias tool developed by Hoy et al. [33] and modified it to suit to our study. The tool consists of ten items that assess sampling, attrition, measurement and reporting bias. The validity of methodology, appropriateness and reporting of results were also assessed (Table 5 in Appendix 2). When the information provided was not adequate to assist in making judgment for a certain item, we agreed to grade that item with a ‘NO’ meaning high risk of bias. Each study was graded depending on the number of items judged ‘YES’ as low (≥ 8), moderate (6 to 7) or high risk of bias (≤ 5) (Table 6 Appendix 2).

The main findings regarding the prevalence and risk factors for GDM were summarized by two authors, and excel sheet was prepared under subheadings agreed upon by all authors. Data were extracted from each study regarding name of author (s), country and sub-region, Study design, setting, year of publication, year of study conducted (year of survey), sample size, response rate, gestational age when GDM screen, participant selection, age of pregnant women, test approach (one step vs two step), screening criteria (Universal vs selective), Blood glucose levels measured by (Glucometer vs Laboratory method), prevalence of GDM (including percentage and 95% CI), odds ratio, relative risk of certain risk factors. The outcome measures extracted were prevalence of GDM and risk factors in terms of differences of proportion/percent of GDM in the total pregnant women were participated.

#### Statistical methods and analysis

The data were entered into Microsoft Excel, exported into STATA/SE version 14 software for analysis. The heterogeneity test of the included studies was assessed by using the *I*^2^ statistics and Q test with its respective *p*-value. The presence of heterogeneity was considered to *I*^2^ test statistics results > 50% [34, 35] and Q test and its respective *P*-value < 0.05. Furthermore, the heterogeneity was presumed in the protocol based on an estimate of a potential variation across studies and depicted in the analyses, we used a random effects model as a method of analysis [34]. The publication bias was assessed using the Egger‘s regression test objectively and funnel plot subjectively [36, 37]. Any asymmetry of a funnel plot and statistical significance of Egger’s regression test (P-value < 0.05) was suggestive of publication bias. Therefore, the Duval and Tweedie nonparametric trim and fill analysis using the random effect analysis was performed [38]. Forest plots used to present the combined prevalence and 95% confidence interval (CI). Subgroup analyses for prevalence were performed by sub regions of Africa, publication year of studies, quality of the study and study design. In addition, a sensitivity analysis was done to point out the study (s) that caused variation. The different factors associated with GDM were presented using odds ratios (ORs) with 95% confidence interval (CI).

## Result

### Description of included studies

**Fig. 1:**
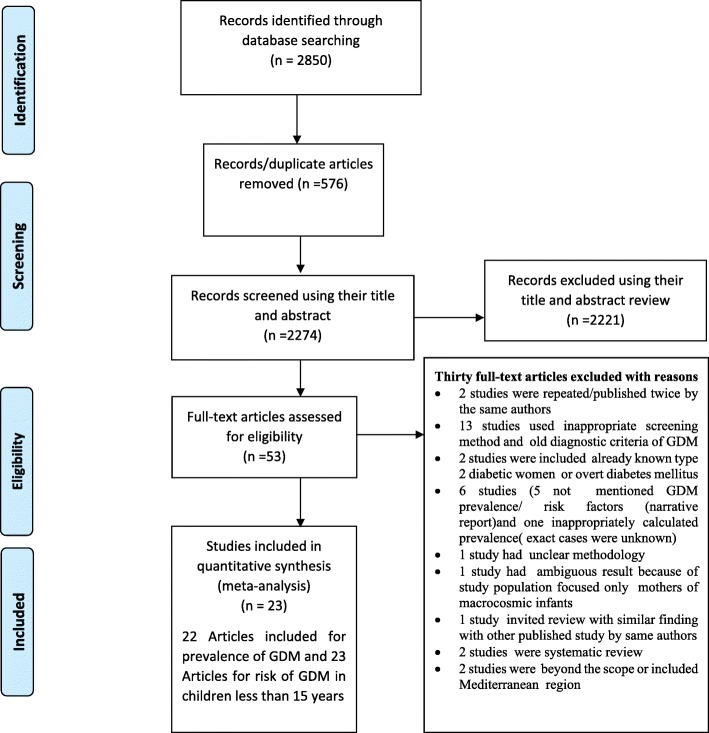
Flow diagram of the included studies for the systematic review and meta-analysis of prevalence and determinants of gestational diabetes mellitus in Africa, 2013–2018

A total of 2,850 published articles were retrieved; of which 576 duplicate records were removed and 2221 records were excluded after screening by title and abstract. A total of 53 full-text articles were screened for eligibility. From those, 30 full-text articles were excluded, because they failed to fulfill the eligibility (*n* = 13) and 17 articles were excluded for reasons for prior criteria. Finally, 23 studies were included in the final analysis (Fig. 1).

### Characteristics of the included studies

Thirteen African countries were represented in this review. Of these, 9 (39.1%) of the studies were from West African [17, 39–46], 6 (26.08%) from East African countries [11, 13, 47–50], 3 (13.04%) from only one Central Africa (Cameroon) [51–53], 3 (13.04%) from Southern Africa [15, 54, 55], and 2 (8.695%) were from only one Northern African country (Egypt) [56, 57]. Regarding the study design, the majority [13] were cross-sectional [11, 13, 40, 41, 43–45, 48, 53–57], nine were cohort [15, 17, 39, 42, 47, 49–52], and one case control study [46]. A total of 11,902 participants were included in the review (Table 1).

**Table 1:** Summary characteristics of studies in the meta-analysis to show the prevalence Gestational diabetes mellitus in Africa, 2013–2018

S.N	Author, year of publication (Year study conducted or year of survey)	Country, Sub region	Study design	Sample size	Response rate	Mean age (SD)/range	GA when tested GDM(week)	Screening criteria	Test approach	Blood glucose levels were measured by	Prevalence of GDM (95% CI)
1	Niyibizi et al., 2016 [13] (2012)	Rwanda, East Africa	Institution-based cross-sectional	96	100%	M = 27 ± 9.8 R = 21–45	24–28	Universal	One step	Glucometer (ACCU-CHECK-Aviva Plus) + laboratory glucose Oxidase method)	8.3% (2.78,13.82)
2	Sagheer and Hamdi, 2018 [56] (2015)	Egypt, North Africa	Institution-based Cross-sectional	700	89.7%	M = 26.5 ± 5.5 R = 18–42	24–28	Universal	One step	Laboratory method	7.43% (5.49,9.37)
3	Ogoudjobi et al., 2017 [44] (2015–2017)	Benin, West Africa	Institution-based cross-sectional	967	100%	M = 28.5 ± 5.7 R = 16–44	24–28	Universal	One step	Laboratory glucose oxidase method	7.5% (5.84.9.16)
4	Oppong et al., 2015 [45](2013)	Ghana, West Africa	Institution-based cross-sectional	399	100%	NR	24–28	Universal	One step	Laboratory method	9.3% (6.45,12.15)
5	Oriji et al., 2017 [41] (2015)	Nigeria, West Africa	Institution-based cross-sectional	235	94%	NR	24–28	Universal	One step	Laboratory glucose oxidase method	14.9% (10.35,19.45)
6	Agbozo et al., 2018 [39] (2016)	Ghana, West Africa	Institution-based Prospective study	435	88.6%	R = 15–54	13–34	Selective (13th–20th) Universal (20th -34th)	One step	NR	9.0% (6.3, 11.69)
7	Nakabuye et al., 2017 [47] (2014)	Uganda, East Africa	Prospective cohort study	251	75.4%	NR	24–36	Universal	One step	Glucose meter (Glucocard™ Σ1070)	30.3% (24.61,35.99)
8	Macaulay et al., 2018 [54] 2013–2017	South African, Southern Africa	Institution-based cross-sectional	1906	94.8%	M = 30 R = 25–35	24–28	Universal	One step	Glucometer(ACCU-CHEK)	9.1% (7.81, 10.39)
9	Abbey and Kasso, 2018 [40] (2016–2017)	Nigeria, West Africa	cross-sectional study	288	> 100%	M = 31.18 ± 4.7	< 14 weeks	Universal	One step	Laboratory glucose oxidase method	21.2% (16.48, 25.92)
10	Njete, John et al., 2018 [48] (2015–2016)	Tanzania, East Africa	Cross-sectional study	333	77%	M = 27.9 ± 5.9	24–28	Universal	One step	Plasma-calibrated hand-held glucometers (GlucoPlus)	19.5% (15.24,23.76)
11	Pastakia et al., 2017 [49] (2013–2015)	Kenya, East Africa	Prospective study	616	71.1%	M = 26.1	24–32	Universal	Two step	Laboratory method and POC tests	2.9% (1.57,4.23)
12	Olagbuji et al., 2017 [42] (2015–2016)	Nigeria, West Africa	Institution-based prospective cohort study	280	NR	R = 18–45	24–32	Universal	Two step	Laboratory glucose oxidase method	15.71% (11.45,19.97)
13	Munang et al., 2017 [51] (2012–2013)	Cameroon, Central Africa	Institution-based prospective study	400	82%	M = 26 ± 5	24–28	Universal	Two step	Glucometer (Accu-Chek® Compact Plus)	32.1%. (27.52,36.68)
14	Jao, Wong et al., 2013 [53] (2013??) NR	Cameroon, Central Africa	Cross Sectional study	316	NR	R = 15–50	24–28	Universal	One step	NR	6.3% (3.62,8.98)
15	Djomhou et al., 2016 [52] (2013)	Cameroon, Central Africa	Institution-based prospective cohort study	100	100%	M = 27 ± 6	All GA	Universal	NR	Laboratory method	22% (13.88,30.12)
16	Olagbuji et al., 2015 [17] (2012–2014)	Nigeria, West Africa	Institution-based prospective study	1059	81.7%	M = 30.7 ± 4.4	24–32	Universal	one-step	Laboratory glucose oxidase method	8.6% (6.91,10.29)
17	Ogu et al., 2017 [43] (2014–2015)	Nigeria, West Africa	Institution-based cross-sectional	837	NR	M = 30.67 ± 4.55 R = 18–48 years	NR	Selective	NR	NR	3.3% (2.09, 4.51)
18	Khalil et al., 2017 [57] (2015–2016)	Egypt, North Africa	Institution-based Cross sectional	250	100%	NR	24–28	Universal	Two step	Laboratory glucose oxidase method	8%. (4.64, 11.36)
19	Adams and Rheeder, 2017 [15] (2012??) NR	South Africa, Southern Africa	Prospective cohort study	554	55.4%	M = 27.2 ± 5.8	24–28	Universal	One step	Laboratory method + POC test	25.8% (22.16, 29.44)
20	Adoyo et al., 2016 [50] (2015)	Kenya, East Africa	Cohort study design	238	93.7	M = 33.06 (GDM) & 27.9 (Non GDM)	≥28	NR	NR	NR	27.73% (22.04,33.42)
21	Mwanri et al., 2013 [11] (2011–2012)	Tanzania, East Africa	cross-sectional study	910	NR	M = 27.5(5.0) Urban & M = 26.6 (5.3) Rural	≥20 Weeks	Universal	One step	POC (HemoCue Glucose B-201)	13.1% (10.91, 15.29)
22	Asare-Anane et al. 2014 [46] 2010.	Ghana West Afrcia	Case control	200	100	NR	All GA	Universal	NR	NR	NA
23	Mathilda et al., 2017 [55] (2010–20140	Zimbabwe. Southern Africa	Institution-based cross-sectional	532	100%	M = 26.9 ± 6.7	All GA	NR	NR	Laboratory method	8.5 (6.13, 10.87)

*GDM* Gestational diabetes mellitus, *M* mean, *R* Range, *SD* Standard deviation, *NR* Not Reported, *GA* Gestational Age, *POC* Point of care, *NA* Not Applicable

Studies were categorized according to their risk of bias; ten studies were considered to have low 12 (52.2%), 4 (17.4%) moderate, and 7 (30.43%) as having high risk of bias (Tables 5 and 6 in Appendix 2). The studies with high risk of bias had either the small sample size, unclear selection of study participants, unclear measurement protocol, low response rate, and/or data collected from hospital records rather than from subjects.

### Prevalence of GDM in Africa

Twenty-two articles included in the meta-analysis to estimate the prevalence of GDM in Africa. A total of 11,702 pregnant women were included in the analysis. The included studies reported sample size which ranged from 96 participants in Rwanda [13] to 1906 in South Africa [54] (Table 1).

**Fig. 2:**
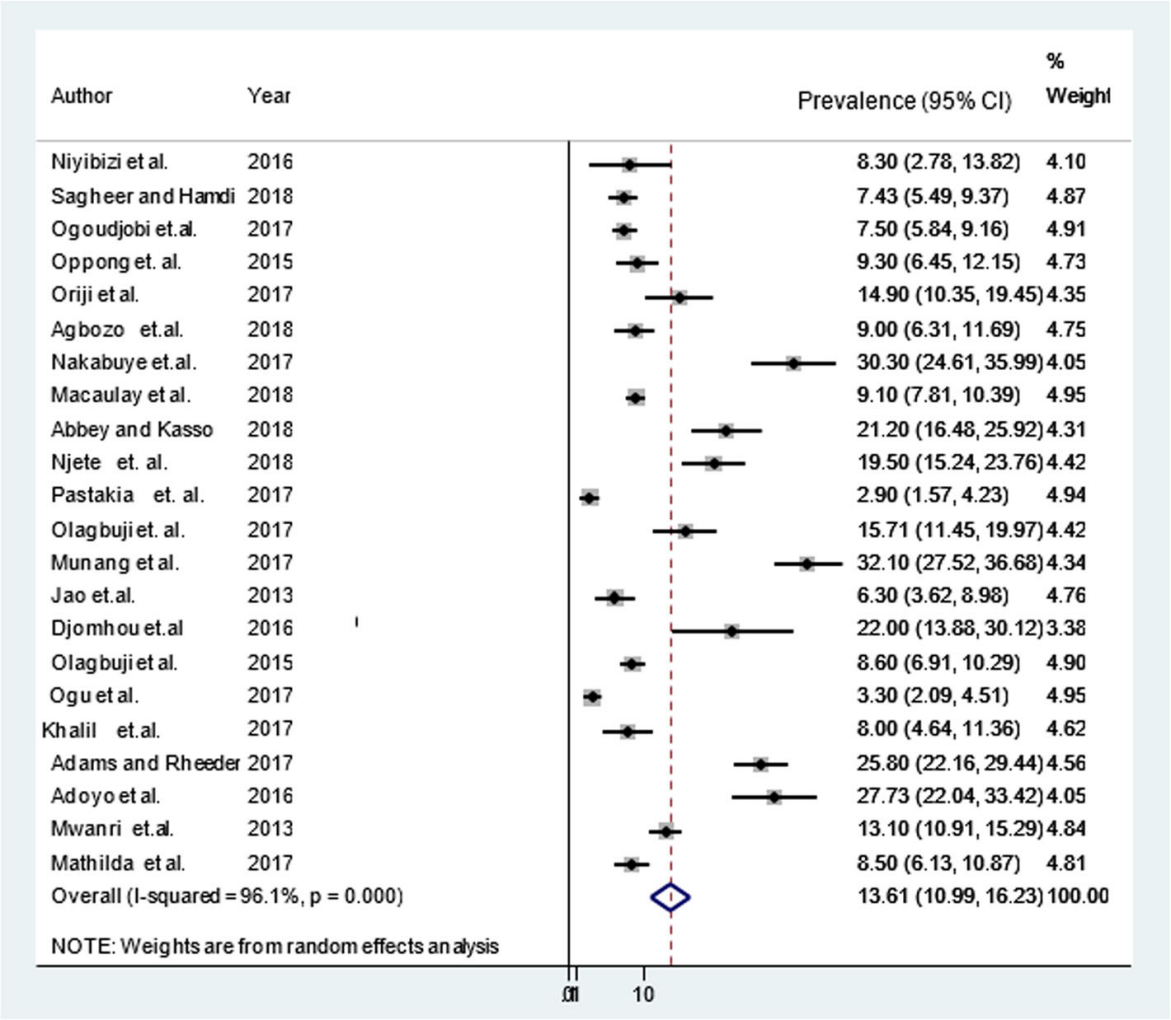
Prevalence of gestational diabetes mellitus in Africa, 2013–2018

The random effect pooled prevalence of GDM in Africa was 13.61% (95% CI: 10.99, 16.23; I^2^ = 96.1%, *p* <  0.001) (Fig. 2). However, there was a publication bias (Egger’s test, βo = 7.98, *p*-value < 0.001). The trim and fill analysis added ten studies and the pooled prevalence of GDM in Africa varied to 6.81% (95% CI: 3.96, 9.7). There was significant heterogeneity (I^2^ = 96.1%) and Q test (Tau-squared = 35.6783, *p*-value *p* <  0.001) in the prevalence of GDM in Africa, which is likely due to differences prevalence in sub regions of Africa, publication year of studies, risk of bias and study design. Therefore, sub-group analysis showed that the pooled prevalence of GDM in sub-Saharan Africa was 14.28% (95% CI: 11.39, 17.16; I^2^ = 96.4%, *p* <  0.001).

Similarly, the sub-group analysis by sub-region showed that the prevalence of GDM was highest, 20.4% (95% CI: 1.55, 38.54) in the Central African sub-region, followed by 16.76% (95% CI: 8.47, 25.05) in East African sub-region, 14.28% (95% CI: 6.22, 22.35) in Southern Africa, 10.72% (95% CI: 7.52, 13.91) in West Africa, and the lowest was in Northern Africa, 7.57% (95% CI: 5.89, 9.25) (Table 2).

**Table 2:** Subgroup analysis of the prevalence of gestational diabetes mellitus in Africa, 2013–2018

Subgroup	Number of studies	Total Sample	Prevalence (95%CI)	Heterogeneity			
				Q-value	Df	I^2^	*P*-value
Sub-region							
East Africa	6	2444	16.76 (8.47, 25.05)	102.242	5	97.6	< 0.001
Southern Africa	3	2992	14.28 (6.22, 22.35)	49.0036	2	97.3	< 0.001
West Africa	8	4500	10.72 (7.52, 13.91)	18.7272	7	93.4	< 0.001
Central Africa	3	816	20.04 (1.55, 38.54)	259.1118	2	97.9	< 0.001
Northern Africa	2	950	7.57 (5.89, 9.25)	0.00	1	0.00	0.774
Sub Saharan African countries	20	10,752	14.28 (11.39, 17.16)	39.6514	19	96.4	< 0.001
Publication year of study							
2013	2	1226	9.75 (3.08, 16.41)	21.5605	1	93.3	< 0.001
2015	2	1458	8.78 (7.33, 10.23)	0.0000	1	0.0	0.679
2016	3	434	19.27 (6.63, 31.91)	113.7686	2	91.7	< 0.001
2017	10	4922	14.55 (9.69, 19.40)	58.1562	9	97.7	< 0.001
2018	5	3662	12.67 (8.78, 16.55)	17.1333	4	91.9	< 0.001
Risk of bias							
Low	12	7488	13.49 (10.08, 16.90)	33.9381	11	96.6	< 0.001
Moderate	3	817	14.56 (3.19, 25.94)	96.6674	2	96.5	< 0.001
High	7	3397	13.77 (8.14, 19.41)	52.1874	6	95.7	< 0.001
Study design							
Cross-sectional study	13	7769	10.14 (7.86, 12.41)	15.0084	12	92.1	< 0.001
Prospective study	9	3933	19.09 (12.35, 25.82)	100.7873	8	97.9	< 0.001

Sub-group analysis by publication year of studies indicated a highest, 19.27% (95% CI: 6.63, 31.91) prevalence of GDM was observed by 2016 and the lowest, 8.78% (95% CI: 7.33, 10.23) by 2015. Relating to the quality score of included studies, the prevalence of GDM in articles of low risk bias, 13.49% (95% CI: 10.08, 16.90), moderate risk bias 14.56% (95% CI: 3.19, 25.94), and high risk of bias (13.77% (95% CI: 8.14, 19.41), were similarly observed. Furthermore, the prevalence of GDM in articles conducted by cross sectional and prospective study design was 10.14% (95% CI: 7.86, 12.41) and 19.09% (95% CI: 12.35, 25.82) respectively (Table 2).

### Factors associated with GDM

#### Demographic characteristics

The demographic factors included in this analysis were maternal age, parity, obesity, and family history of DM. A separate analysis was conducted for each variable. A total of 13 articles [13, 17, 40, 41, 43–45, 47, 48, 50, 54, 56, 57] were included to determine the association of maternal age and GDM. Seven out of 13 studies [17, 41, 44, 48, 50, 54, 57] had a significant association between maternal age and GDM, while the other six articles [13, 40, 43, 45, 47, 56] showed non-significant associations. In the random-effect model, the pooled odds of GDM among women aged ≥30 years is increased by nearly three folds (OR = 2.83; 95% CI: 1.75, 4.59: The I^2^ = 90.5%, *p*-value < 0.001). The sensitivity analysis showed that there is no influential study that caused variation, however, there was a publication bias (Egger’s test, βo = 2.64; *p*-value = 0.004). The final pooled effect size by the trim and fill analysis by added seven studies showed that there was no significant association between maternal age and GDM, OR = 1.27 (95% CI = 0.81, 1.99, *p*-value = 0.297). Moreover, a total of 11 articles [40, 41, 43–48, 54, 56, 57] were included to determine the association of multi-parity and GDM, and only five of the studies [43, 46–48, 57] had a significant association with GDM. There was heterogeneity (I^2^ = 79.3%, *p*-value < 0.001) among subgroups and no influential study caused variations by the sensitivity analysis. There was a publication bias (Egger’s test, βo = 3.9; *p*-value = 0.004). However, the pooled effect size by the trim and fill analysis by added six studies showed that there was no significant association between multi parity (≥ 2) and GDM, OR = 1.09 (95% CI = 0.63, 1.90, *p*-value = 0.758) (Table 3).

**Table 3:** Summary of the meta-analysis of associated factors for gestational diabetes mellitus in Africa, 2013–2018

No.	Factors	No of studies	OR (95% CI)	*P* value	Heterogeneity				Publication bias (Egger’s test)	Range of result of by omitted one study at a time	
					Q-value	Df (Q)	*p* –value	I^2^	*p*- value	Minimum	Maximum
1	Maternal age (≥ 30 year)	13	1.27 (0.810, 1.992)	0.297	249.831	19	< 0.001	90.5%	0.004	0.56	1.12
2	Maternal overweight and/obesity	12	3.51 (1.92, 6.40)	0.005	146.455	12	< 0.001	88.4%	0.231	0.65	1.86
3	Parity (≥ 2)	11	1.091 (0.628, 1.897)	0.758	121.267	16	< 0.001	79.3%	0.004	0.34	1.39
4	Having macrosomic baby	10	2.23 (1.12,4.44)	0.023	55.883	10	< 0.001	76.8%	0.017	0.42	1.65
5	Family history of diabetes	13	2.69 (1.84, 3.91)	0.005	84.227	16	< 0.001	70.0%	0.143	0.61	1.36
6	History of still birth	4	2.92 (1.23, 6.93)	0.015	12.751	3	0.005	76.5%	0.742	0.20	1.94
7	History of abortion	8	2.21 (1.68, 2.92)	0.000	13.285	8	0.102	35.9%	0.985	0.52	1.07
8	History of hypertension	9	2.49 (1.35, 4.59)	0.004	31.043	8	< 0.001	74.2%	0.952	0.30	1.52
9	Previous history of GDM	3	14.16 (2.39, 84.08)	0.004	5.619	2	0.060	64.4%	0.128	0.87	4.43

**Fig. 3:**
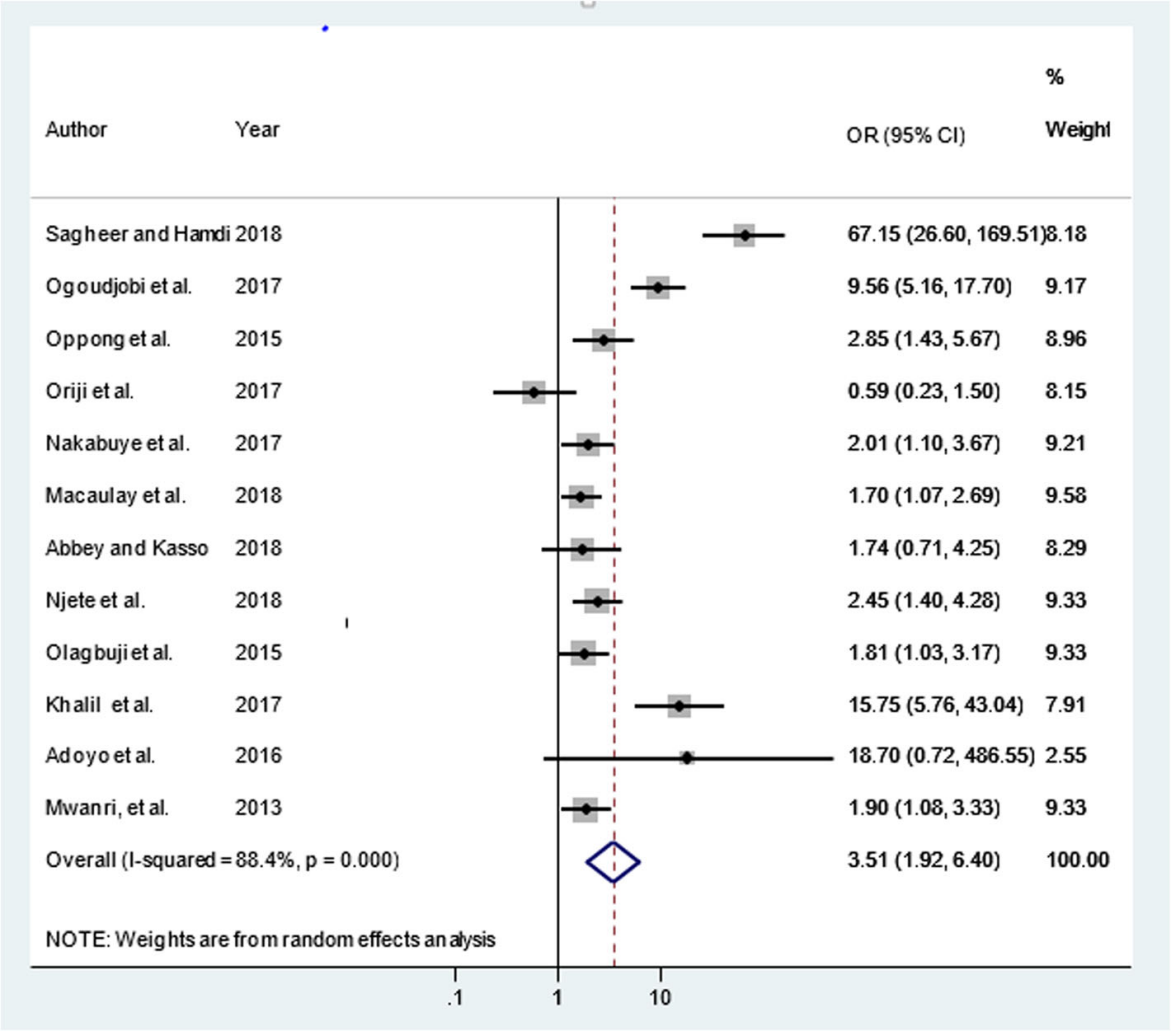
Maternal overweight and/ obesity and gestational diabetes mellitus in Africa, 2013–2018

**Fig. 4:**
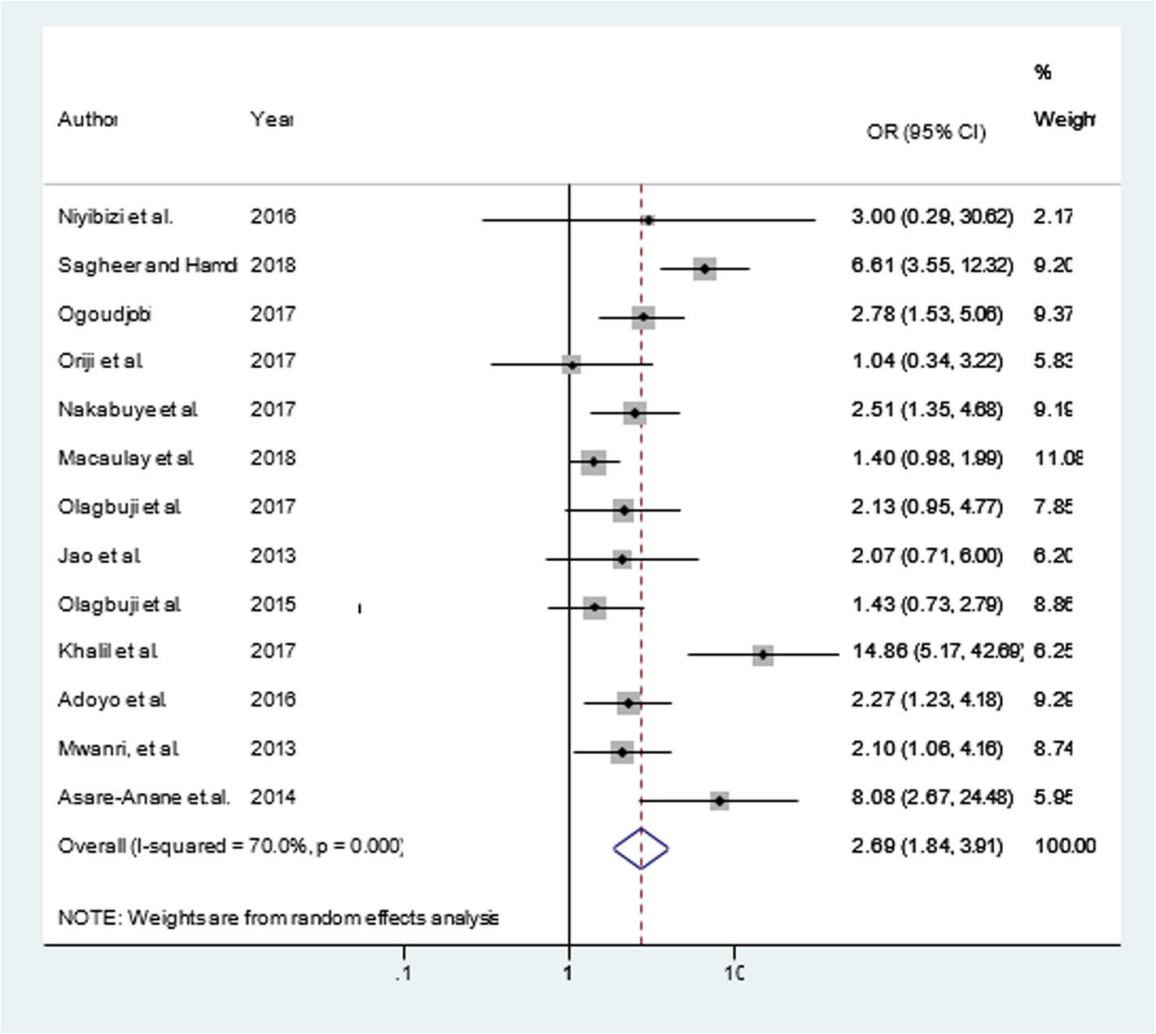
Family history of diabetes mellitus and gestational diabetes mellitus in Africa, 2013–2018

Similarly, a total of 12 articles [11, 40–42, 44, 45, 47, 48, 50, 54, 56, 57] were included to determine the association of maternal overweight and/or obesity and GDM. Nine of the included studies [11, 17, 44, 45, 47, 48, 54, 56, 57] had significant association while the rest [40, 41, 50] showed a non-significant association between maternal overweight and/or obesity and GDM. Even though, there was heterogeneity (I^2^ = 88.4%, *p*-value < 0.001) among subgroups, there was no influential study that caused variation by sensitivity analysis and no publication bias (Egger’s test, βo = 3.22; *p*-value = 0.231). The final pooled effect size showed that pregnant women with maternal overweight and/or obesity were more than three times (OR = 3.51; 95% CI = 1.92, 6.40) more likely to increase the risk of GDM (Fig. 3). Additionally, out of a total of 13 articles [11, 13, 17, 41, 42, 44, 46, 47, 50, 53, 54, 56, 57] included to determine the association of family history of diabetes mellitus and GDM seven of them [11, 44, 46, 47, 50, 56, 57] had significant association with GDM. The pooled odds of developing GDM was (OR = 2.69; 95% CI = 1.84, 3.91: I^2^ = 70%, *p*-value < 0.001), there was no any influential study that caused variations by the sensitivity analysis and no publication bias was observed (Egger’s test, βo = 1.965; *p*-value =0.143). The random-effect analysis showed that family history of diabetes mellitus were nearly three times more likely increased the risk of GDM (Fig. 4).

#### Medical factors

The medical factors included in this analysis were chronic hypertension and history of previous GDM. A total of 9 articles [15, 43–45, 48, 50, 56, 57] were included to see the association of history of chronic hypertension and GDM, of which 4 of them [15, 44, 50, 56] have shown a significant association with GDM. In the random-effect model, the overall odds of developing GDM among women suffered from chronic hypertension was raised by 2.5 folds (OR = 2.49; 95% CI = 1.35, 4.59: (I^2^ = 74.2%, *p*-value < 0.001) than their counterparts. According to the sensitivity analysis, there was no any influential study that causes variation. Likewise, publication bias was not a concern (Egger’s test, βo = − 0.096; *p*-value =0.952). Moreover, three articles [41, 45, 56] were included to determine the association of the history of previous GDM on the current risk of GDM, and one study [41] showed that it has a significant positive association. The pooled odds of GDM with women experienced in GDM in the previous times was increased by (OR = 14.16; 95% CI = 2.39, 84.08: (I^2^ = 64.4%, *p* = 0.060)). There was the absence of an influential study that contributed to the variation. No publication bias was observed (Egger’s test, βo = − 3.95; *p*-value = 0.128). Therefore, the final pooled effect size showed that having the history of previous GDM were fourteen times more likely increased the risk of GDM (Table 3).

#### Obstetric factors

**Fig. 5:**
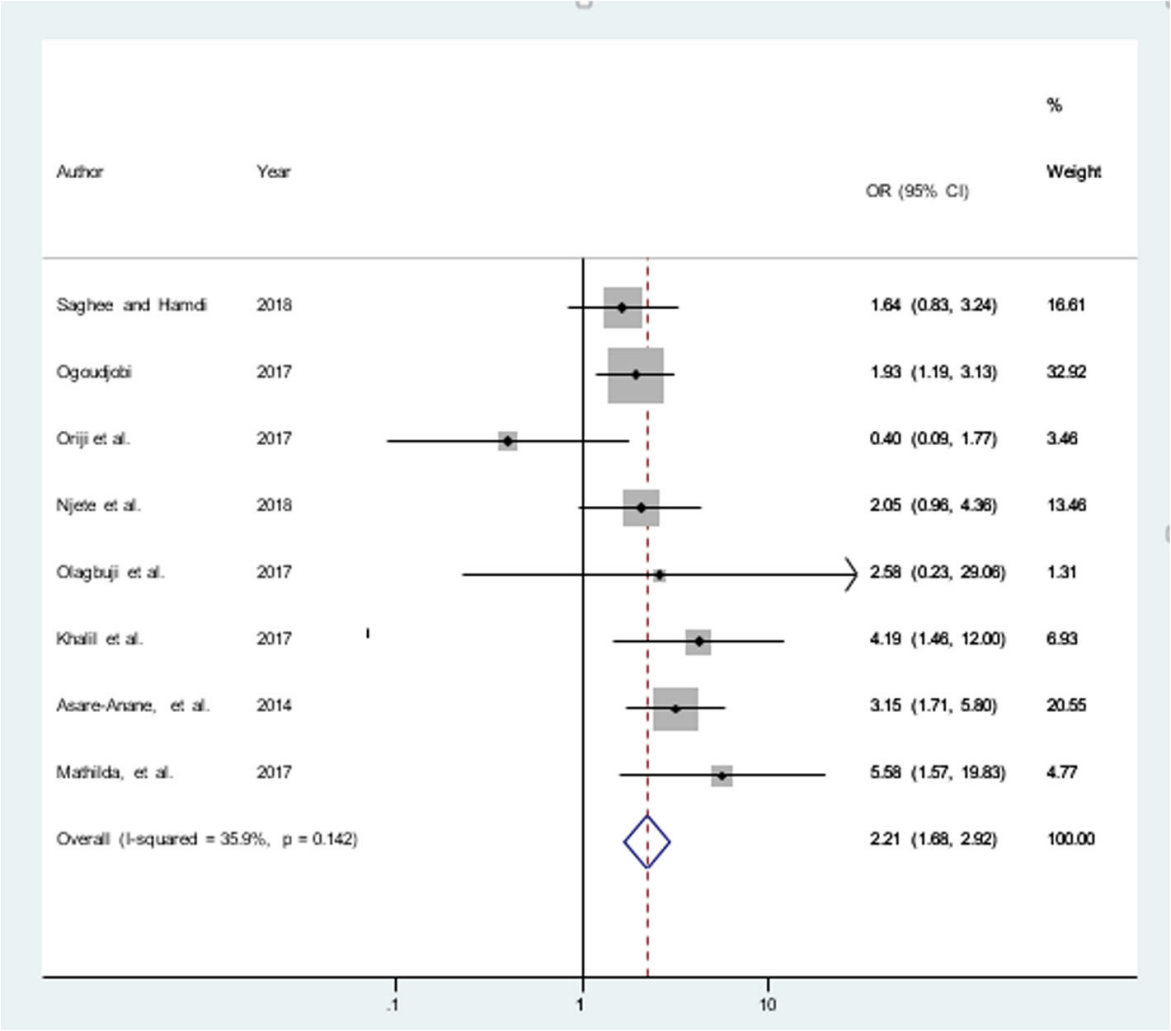
History of abortion and gestational diabetes mellitus in Africa, 2013–2018

The obstetric factors included in this analysis were having history macrosomia (large size baby), stillbirth, and abortion or miscarriage. A total of 10 articles [17, 40–42, 44, 47, 48, 54, 55, 57] were included to determine the association of macrosomia and GDM, and half of them found a positive association [40, 44, 55, 57]. In the random-effect model, the pooled odds of experiencing GDM among women who gave birth to the macrocosmic neonate in former pregnancy was raised by nearly three times (OR = 2.81; 95% CI = 1.52, 5.21: I^2^ = 76.8%, *p* <  0.001). The sensitivity analysis revealed that no influential study that resulted in variation. Additionally, there was a publication bias (Egger’s test, βo = 6.46; *p*-value = 0.017), and the trim and fill analysis by added one study highlighted that women who gave birth to macrocosmic baby were two times more likely to develop GDM as compared to their counterparts, OR = 2.23 (95% CI = 1.12, 4.44, *p*-value = 0.023) (Table 3). Additionally, a total of four articles [11, 41, 44, 46] were included to determine the association of history of stillbirth and GDM, and only one of the study [41] showed a non-significant association. In the random-effect model, the overall odds of GDM among women having history of stillbirth was three times (OR = 2.92; 95% CI = 1.23, 6.93: I^2^ = 76.5%, *p* <  0.001). There was no any influential study that caused variation according to the sensitivity analysis and no publication bias was observed (Egger’s test, βo = − 1.93; *p*-value =0.742) (Table 3). Furthermore, eight articles [41, 42, 44, 46, 48, 55–57] were included to determine the association of history of abortion and GDM, and half of them [44, 46, 55, 57] showed a positive association. The overall odds of GDM among women having abortion history was two times (OR = 2.21; 95% CI = 1.68, 2.92: I2 = 35.9%, *p* = 0.142), and no publication bias was detected (Egger’s test, βo = 0.233; *p*-value = 0.985) (Fig. 5). Moreover, supplementary files on original funnel plots and funnel plots improved by the trim and fill method also presented (Additional file 1).

## Discussion

This systematic review and meta-analysis was conducted to estimate the prevalence and determinants of GDM in Africa using the updated and current international diagnostic criteria for GDM. The IDF reports GDM is a severe and neglected threat to women and their offspring [4].

The pooled prevalence of GDM in Africa was 13.61% (95% CI: 10.99, 16.23) with a higher estimate compared to other low and middle-income countries (LMIC). However, it was varied to 6.81% (95% CI: 3.96, 9.7) by added ten studies in the trim and fill analysis. High prevalence of GDM were reported in some included studies in the this meta-analysis for instance in Cameron (32.1%) [51] and Uganda (30.3%) [47]. It might be due to these studies were universal screening strategy for all pregnant woman after 24 weeks of gestation. Selective screening strategy misses up to 45% of mothers with GDM [58, 59].

In this meta-analysis the pooled prevalence of GDM in sub-Saharan Africa was 14.28%, which depicts a public health concern. The finding is higher as compared to the finding of a previous systematic review conducted in sub-Saharan Africa (5.1%) [9], Africa (6.0%) [26], Europe (5.4%) [7], Asia (11.5%) [8], and Eastern and Southeastern Asia (10.1%) [18]. Similarly, it’s greater than those whose results were reported in Western countries, including Europe, US, and Australia, reporting the prevalence of 5.4, 9.2, and 5.7%, respectively [7, 60, 61]. This could be because of the use of different diagnostic criteria and the lack of consensus regarding the use of diagnostic criteria for GDM which might have largely contributed to the heterogeneity of GDM prevalence. In addition, the discrepancies could be related to a higher detection rate in recent years due to improved diagnosis of GDM during pregnancy at earlier gestational age and increased access to these tests more than before.

The high heterogeneity in the overall prevalence seen in our study may be due to several reasons. As the result we considered post-hoc subgroup analyses by different characteristics such as sub- regions of Africa, publication year of study, study quality, and study design. Variations in the rate of GDM were observed in different sub-regions of Africa, the highest in Central Africa (20.4%) and the lowest in Northern Africa (7.57%). This discrepancy might be attributable to sociocultural, environmental, and economic factors, resulting in differences in accessing ANC services. These factors were also mentioned as one of the reasons for a high level of GDM in sub-Saharan Africa. Moreover, the sub-group analysis found GDM prevalence with publication year of studies a highest by 2016 (19.3%) and the lowest by 2015(8.8%), relating to the quality of study score low risk bias (13.5%), moderate risk bias(14.6%), and high risk of bias (13.8%) which were similarly observed. In the same way, studies conducted by cross sectional and prospective study design found that overall pooled prevalence of GDM were 10.14 and 19.1% respectively. However still significant heterogeneity was also found in sub group analyses and these differences between groups may not statistically be reliable as the CIs overlap. This was further augmented by further supplementary analysis that revealed non-significant group differences.

This review also assessed the association of GDM with overweight and obesity, those who had a macrocosmic baby, family history of diabetes mellitus, history of stillbirth, history of abortion or miscarriage, chronic hypertension, and previous history of GDM in Africa.

Pregnant women with obesity had increased risk of GDM than pregnant women with normal weight (OR = 3.51; 95% CI = 1.92, 6.40). The finding was consistent with those of the previous meta-analysis conducted in sub-Saharan Africa [9], US [62], the Hyperglycemia and Adverse Pregnancy Outcome Study (HAPO) [63], a systematic review and meta-analysis by Nelson SM et al. which revealed [64] pre-pregnancy BMI was more strongly associated with the risk for GDM. The possible reason could be GDM due to the reduction of insulin sensitivity among obese pregnancies. In other words, obesity-related insulin resistance inflates the normal glucose levels [64, 65]. Moreover, it might be because women with overweight or obesity might be exposed to a sedentary life, again they became obese due to inactive in their daily activities. The present result has public health implications given the increasing prevalence of obesity; we can also expect a further rise in the prevalence of GDM in the coming years.

The study showed that having a family history of diabetes mellitus was a significant factor for an increased risk for GDM (OR = 2.69; 95% CI = 1.84, 3.91). The finding was consistent with Carr DB et al. study in the US [66], and Iran [67, 68]. This is utterly known that if the beta cell is not functional genetically, hyper-glycaemia will occur linked with insulin resistant surely happen; this is especially true family suffering of type I diabetes mellitus and due to the familial tendency of insulin secretory defects [69].

The current meta-analysis also found that pregnant women with a previous history of GDM had fourteen times higher risk of developing GDM in the future pregnancy (OR = 14.16; 95% CI = 2.39, 84.08). This finding was in line with findings from Colorado [70], a systematic review conducted by Catherine Kim et al. [71]. The recurring nature of GDM because of the shared risk factor in repeated pregnancies [72]. Similarly, this study also found that the odds of GDM were 2.5 times higher in women with chronic hypertension (OR = 2.49; 95% CI = 1.35, 4.59). This finding agrees with findings from Canada and India [66, 73, 74]. This might be due to the potential complication like obesity, inflammation, oxidative stress, insulin resistance, and mental stress owing to chronic hypertension could lead to GDM [75].

The current meta-analysis also found that pregnant women who were ever had macrocosmic (large sized) baby was more likely to develop GDM compared to their counterparts. Nearly similar finding observed by random effect model (OR = 2.81; 95% CI = 1.52, 5.21) and by added one study in the trim and fill analysis (OR = 2.23; 95% CI = 1.12, 4.44). The finding was consistent with the HAPO study [76], a cohort study in Nova Scotia Atlee Perinatal Database (NSAPD) in Canada by Stephanie et al. [77]. This could be due because large infant birth weight during index pregnancy may be indicative of poor control and/or poor maternal diet or may reflect GDM severity, which might predispose the women to recurrent GDM [77].

Moreover, women with a history of stillbirth had three times (OR = 2.92; 95% CI = 1.23, 6.93) higher risk of developing GDM in future pregnancies. The finding was in line with a review conducted in sub-Saharan Africa by Mwanri et al. [9]. Similarly, women with a history of miscarriage or abortion had two folds of higher risk for developing GDM (OR = 2.21; 95% CI = 1.68, 2.92). This finding agrees with findings from Australia [78] and China [79]. The risk of stillbirth and abortion or miscarriage during the index pregnancy might be indicative of women with poor blood glucose control and various endocrine system problems which affect the normal metabolism of insulin, which might then predispose women to recurrent or risk for GDM.

This review has certain strengths and limitations. This resulted the pooled prevalence of GDM noted using the updated and current international diagnostic criteria and using a similar definition of GDM allows to determine the current and true prevalence of GDM in Africa. Subgroup analysis (sub-regions of Africa, publication year of studies, risk of bias and study design) and assessed multiple factors were considered as the strength of our study. However, our meta-analysis has limitations, such as the presence of significant heterogeneity, only studies published in English included, did not used MeSH terms in the search strategy, and did not investigate grey literature. Hence the result of this meta-analysis had significant heterogeneity and there was some overlap of CIs in the result of sub group analysis. Therefore, some estimates could be influenced by an interaction between groups. Although, most of the articles included in this review assessed the demographic characteristics, medical factors, and obstetric factors, there were limited studies which presented the association of other variables like residence, dietary diversity, substance abuse, and physical activity issues with GDM. Future review studies which elucidate the association of GDM with other factors listed above. Additionally, this review didn’t include qualitative studies on the reasons for GDM.

## Conclusions

The prevalence of GDM was found to be high in Africa. Wide differences in the prevalence of GDM were observed across the different sub-regions of Africa, the highest being in Central Africa and the East Africa regions. The prevalence was high in the sub-Sharan Africa region. The associated factors for GDM include women with obesity and overweight, previous fetal macrosomia, family history of diabetes, history of abortion/miscarriage, history of stillbirth, chronic hypertension, and history of previous GDM. Therefore, considering the trend towards the epidemic of obesity, a substantial burden of GDM is anticipated in Africa. Preventing overweighed and obese, giving due attention for women having family history of DM, poor obstetric history and women with history of previous GDM are strongly recommended to mitigate the burden. It is essential to further identify effective modifiable factors, and early screening and diagnosis of GDM for better management and to halt the burden.

### Additional file

Additional file 1: Supplementary files on original funnel plots and funnel plots improved by the trim and fill method. (DOCX 162 kb)

## Data Availability

All data pertaining to this study are contained and presented in this document.

## Appendix 1

**Table 4:** Search terms used for final search 26 November, 2018

Data based used	Search term	Items found	Date and Time
PubMed	(((diabetes OR hyperglycemia OR "glucose intolerance" OR "gestational diabetes" OR "impaired glucose tolerance" OR "impaired fasting glucose" OR "diabetes mellitus" OR "postprandial glucose tolerance" OR "glucose tolerance")) AND (("physical inactivity" OR "sedentary life style" OR "physical activity" OR "previous foetal macrosmia" OR "pervious unexplained still birth" OR "previous still birth" OR "family history of type 2 diabetes mellitus" OR "high mid upper arm circumference" OR obesity OR overweight OR "body mass index" OR "previous gestational diabetes mellitus" OR age OR "advanced maternal age" OR "previous pregnancies" OR "polyhydramnios" OR glycosuria OR "depression" OR "dietary diversity" OR HIV OR residence OR "Urban residence" OR "rural residence")) AND (((pregnan*) OR gestation*) OR Gravid*) OR gestational diabetes AND ((Africa* OR east Africa* OR north Africa* OR central Africa* OR Angola* OR algeria OR Benin* OR Botswan* OR burkina faso OR Burkinabe* OR burundi OR Cameroon* OR cape Verde* OR central african republic OR Chad* OR Comor* OR Congo* OR congo, democratic republic of OR cote d'ivoire OR ivory coast OR ivorian OR Djibouti* OR Dominica* OR egypt OR equatorial guinea OR Ecuador* OR Guinea* OR Eritrea* OR Ethiopia* OR Gabon* OR Gambia* OR Ghana* OR guinea OR guinea-bissau OR Kenya* OR lesotho OR Liberia* OR libya OR Madagascar* OR Malawi* OR Mali* OR Mauritania* OR mauritius OR mozambique OR morocco OR Namibia* OR Niger* OR nigeria OR Rwanda* OR sao tome and principe OR Senegal* OR seychelles OR sierra Leone* OR Somalia* OR south Africa* OR south sudan OR Sudan* OR swaziland OR swazi OR Tanzania* OR Togo* OR Tonga* OR Uganda* OR Zambia* OR tunisia OR Zimbabwe*)) AND human NOT animal	1378	17/11/2018 Time 11:57 PM
Scopus	( ALL ( diabetes OR hyperglycemia OR "glucose intolerance" OR "gestational diabetes" OR "impaired glucose tolerance" OR "impaired fasting glucose" ) OR TITLE-ABS-KEY ( "diabetes mellitus" OR "postprandial glucose tolerance" OR "glucose tolerance" ) AND TITLE-ABS-KEY ( "physical inactivity" OR "sedentary life style" OR macrosmia OR "previous still birth" OR "family history of diabetes mellitus" ) OR TITLE-ABS-KEY ( "high mid upper arm circumference" OR obesity OR overweight OR "body mass index" OR "previous gestational diabetes mellitus" OR " maternal age" OR depression OR "dietary diversity" OR residence ) AND TITLE-ABS-KEY ( pregnan* OR gestation* OR gravid* OR "gestational diabetes" ) AND TITLE-ABS-KEY ( africa* OR east AND africa* OR north AND africa* OR central AND africa ) OR TITLE-ABS-KEY ( angola* OR algeria OR benin* OR botswan* OR burkina AND faso OR burkinabe* OR burundi ) OR TITLE-ABS-KEY ( cameroon* OR cape AND verde* OR 'central AND african AND republic' OR chad* OR comor* OR congo* OR congo, AND democratic AND republic AND of OR cote AND d'ivoire OR ivory AND coast OR ivorian ) OR TITLE-ABS-KEY ( djibouti* OR dominica* OR egypt OR equatorial AND guinea OR ecuador* OR guinea* OR eritrea* OR ethiopia* ) OR TITLE-ABS-KEY ( gabon* OR gambia* OR ghana* OR guinea OR guinea-bissau OR kenya* OR lesotho OR liberia* OR libya OR madagascar* OR malawi* OR mali* OR mauritania* OR mauritius OR mozambique OR morocco ) OR TITLE-ABS-KEY ( namibia* OR niger* OR nigeria OR rwanda* OR sao AND tome AND principe OR senegal* OR seychelles OR sierra AND leone* OR somalia* OR south AND africa ) OR TITLE-ABS-KEY ( south AND sudan OR sudan* OR swaziland OR swazi OR tanzania* OR togo* OR tonga* OR uganda* OR zambia* OR tunisia OR zimbabwe* ) AND TITLE-ABS-KEY ( human ) )	543	17/11/2018 Time 12:08 AM
Google scholar	“ Gestational diabetes mellitus OR Hyperglycemia ” AND “ Name each African countries ”	854	26/11/2018 Time 4:22 pm
Other sources	“gestational diabetes” and Africa; “impaired fasting glucose” and pregnancy and Africa; diabetes and pregnancy and Africa; “impaired glucose tolerance” and pregnancy and Africa; “gestational diabetes” and “African countries.”	53	26 /11/2018

## Appendix 2

**Table 5:** Risk of bias assessment tool: Adapted from the Risk of Bias Tool for Prevalence Studies developed by [33] Name of the author and year of publication

Risk of Bias Item	Answer: Yes (Low Risk) or No (High risk)
External Validity	
1. Was the study target population a close representation of the national pregnant population in relation to relevant variables?	
2. Was the sampling frame a true or close representation of the target population? (risk factors used appropriate?)	
3. Was some form of random selection used to select the sample, OR, was a census undertaken?	
4. Was the likelihood of non-participation bias minimal? (i.e. ≥75% response rate)?	
Internal Validity	
5. Were data collected directly from the subjects? (as opposed to medical records)	
6. Were acceptable diagnostic criteria for GDM used?	
7. Was a reliable and accepted method of testing for blood glucose utilised?	
8. Was the same mode of data collection used for all subjects?	
9. Was GDM tested for within the advised gestational period of 24–28 weeks?	
10. Were the numerator(s) and denominator(s) for the calculation of the prevalence of GDM appropriate?	
Summary item on the overall risk of study bias	
A. LOW RISK OF BIAS: 8 or more ‘yes’ answers. Further research is very unlikely to change our confidence in the estimate. B. MODERATE RISK OF BIAS: 6 to 7 ‘yes’ answers. Further research is likely to have an important impact on our confidence in the estimate and may change the estimate. C. HIGH RISK OF BIAS: 5 or fewer ‘yes’ answers. Further research is very likely to have an important impact on our confidence in the estimate and is likely to change the estimate.	

**Table 6:** Summary characteristics of studies in the meta-analysis to show assessment of risk of bias of the included studies the prevalence Gestational diabetes mellitus in Africa, 2013–2018

S.N	Author, year of publication	Sample of pregnant women representative?	Sampling Frame representative?	Random or census selection?	Response rate ≥75%?	Data from subjects?	Acceptable diagnostic criteria?	Reliable testing for BG?	Same method for all?	Proper gestational age GDM test?	Proper calculation prevalence	Total ‘Yes’	Overall risk of bias
1	Niyibizi et al., 2016 [13]	Yes	No	No	Yes	Yes	No	No	Yes	Yes	No	5	High
2	Sagheer and Hamdi , 2018 [56]	Yes	Yes	No	Yes	Yes	Yes	Yes	Yes	Yes	Yes	9	Low
3	Ogoudjobi et al., 2017 [44]	Yes	Yes	No	Yes	Yes	Yes	Yes	Yes	Yes	Yes	9	Low
4	Oppong et al., 2015 [45]	Yes	Yes	No	Yes	Yes	Yes	Yes	Yes	Yes	Yes	9	Low
5	Oriji et.al., 2017 [41]	Yes	No	No	Yes	Yes	Yes	Yes	Yes	Yes	Yes	8	Low
6	Agbozo et al., 2018 [39]	No	No	No	Yes	Yes	No	No	No	Yes	Yes	9	High
7	Nakabuye et al., 2017 [47]	No	Yes	No	Yes	Yes	Yes	No	Yes	Yes	No	6	Moderate
8	Macaulay et al., 2018 [54]	Yes	Yes	No	Yes	Yes	Yes	Yes	Yes	Yes	Yes	9	Low
9	Abbey and Kasso , 2018 [40]	No	No	No	Yes	Yes	No	No	Yes	No	Yes	4	High
10	Njete et al., 2018 [48]	Yes	Yes	Yes	Yes	Yes	Yes	Yes	Yes	Yes	Yes	10	Low
11	Pastakia et al., 2017 [49]	Yes	Yes	Yes	No	Yes	Yes	Yes	Yes	Yes	Yes	9	Low
12	Olagbuji et al., 2017 [42]	Yes	Yes	Yes	Yes	Yes	Yes	Yes	No	Yes	No	8	Low
13	Munang et al., 2017 [51]	Yes	Yes	Yes	Yes	Yes	Yes	No	No	Yes	Yes	8	Low
14	Jao et al., 2013 [53]	No	No	Yes	NR	Yes	Yes	Yes	Yes	Yes	Yes	7	Moderate
15	Djomhou et al., 2016 [52]	No	No	Yes	Yes	Yes	No	No	Yes	No	No	4	High
16	Olagbuji et al., 2015 [17]	Yes	Yes	Yes	Yes	Yes	Yes	Yes	Yes	Yes	Yes	10	Low
17	Ogu et al., 2017 [43]	No	No	No	NR	No	Yes	Yes	Yes	No	Yes	4	High
18	Khalil et al, 2017 [57]	No	No	Yes	Yes	Yes	Yes	Yes	No	Yes	Yes	7	Moderate
19	Adams and Rheeder, 2017 [15]	Yes	Yes	Yes	No	Yes	Yes	Yes	Yes	Yes	Yes	9	Low
20	Adoyo et al., 2016 [50]	No	No	No	Yes	Yes	NR	NR	NR	Yes	Yes	4	High
21	Mwanri et al., 2014 [11]	Yes	Yes	Yes	Yes	Yes	No	Yes	Yes	Yes	No	8	Low
22	Asare-Anane et al., 2014 [46]	Yes	No	No	Yes	Yes	Yes	Yes	No	Yes	NR	6	Moderate
23	Mathilda et al.,2017 [55]	Yes	Yes	No	Yes	No	No	No	No	Yes	No	4	High

*GDM* Gestational diabetes mellitus, *NR* Not Reported

## Abbreviations

BMI: Body mass index
CI: Confidence interval
CM: Centi meter
DM: Diabetes mellitus
FBG: Fasting blood glucose
GDM: Gestational diabetes mellitus
HAPO: Hyperglycemia and Adverse Pregnancy Outcome
I^2^: I-squared statistic
IADPSG: International Association of the Diabetes and Pregnancy Study Group
IDF: International Diabetes Federation
IGT: Impaired glucose tolerance
LMIC: Low and Middle-Income Countries
NCD: Non-Communicable Diseases
OGTT: Oral glucose tolerance test
OR: Odds Ratio
PRISMA: Preferred Reporting Items for Systematic reviews and Meta-analyses guidelines
UN: United Nations
US: United States
WHO: World Health Organization
