# Supplementary material for: Prevalence and determinants of gestational diabetes mellitus in Africa based on the updated international diagnostic criteria: a systematic review and meta-analysis
Source: Arch Public Health. 2019 Aug 6;77:36. doi: 10.1186/s13690-019-0362-0 (PMC6683510; doi:10.1186/s13690-019-0362-0)
Supplement: Supplementary file 1 — Supplementary files on original funnel plots and funnel plots improved by the trim and fill method. (DOCX 162 kb) [file 13690_2019_362_MOESM1_ESM.docx]

**Supplementary files on original funnel plots and funnel plots improved by the trim and fill method.**

**Prevalence of GDM**

**Sensitivity analysis**

**Funnel plot**

**Trim and Fill analysis**

**Factors associated with GDM**

1. **Maternal age and GDM**

**Sensitivity analysis**

**Funnel plot**

**Trim and Fill analysis**

1. **Maternal overweight and/or obesity and GDM**

**Sensitivity analysis**

**Funnel plot**

**Trim and Fill analysis**

1. **Multi parity and GDM**

**Sensitivity analysis**

**Funnel plot**

**Trim and Fill analysis**

1. **Macrosomia and GDM**

**Sensitivity analysis**

**Funnel plot**

**Trim and Fill analysis**

1. **Family history of DM and GDM**

**Sensitivity analysis**

**Funnel plot**

**Trim and Fill analysis**

1. **History of still birth and GDM**

**Sensitivity analysis**

**Funnel plot**

**Trim and Fill analysis**

1. **History of abortion and GDM**

**Sensitivity analysis**

**Funnel plot**

**Trim and Fill analysis**

1. **History of hypertension and GDM**

**Sensitivity analysis**

**Funnel plot**

**Trim and Fill analysis**

1. **History of previous GDM and GDM**

**Sensitivity analysis**

**Funnel plot**

**Trim and Fill analysis**
